# Local re-activation of osteoclast differentiation as a novel therapeutic strategy for osteonecrosis of the jaw

**DOI:** 10.3389/fendo.2024.1447314

**Published:** 2024-07-19

**Authors:** Tommaso Zanocco-Marani, Silvia Ricchiuto, Lorenzo Caselli, Eleonora Lorenzi, Elia Lettucci, Alexis Grande

**Affiliations:** ^1^ Department of Life Sciences, University of Modena and Reggio Emilia, Modena, Italy; ^2^ Department of Biomedical, Metabolic and Neural Sciences, University of Modena and Reggio Emilia, Modena, Italy

**Keywords:** “osteonecrosis of the jaw”, osteoclastogenesis, bisphosphonates, magnesium, differentiation, ONJ, osteoclast

## Introduction

Bisphosphonates are a class of medications primarily used to treat metabolic bone diseases, particularly osteoporosis and Paget’s disease of bone, and neoplastic diseases such as bone metastases deriving from breast or prostate cancer and multiple myeloma. They work by inhibiting bone resorption (1), the process by which bone is broken down and minerals are released into the bloodstream. Bisphosphonates are typically taken orally, although some formulations can be administered intravenously. By reducing bone turnover, bisphosphonates can help to increase bone density, alleviate pain, lower the risk of skeletal-related events (such as fractures or spinal cord compression), and improve the quality of life for patients. In addition to their anti-resorptive role, bisphosphonates have other potential benefits in cancer therapy, in fact some research suggests that bisphosphonates may have anti-tumor effects, such as inhibiting the growth and spread of cancer cells (2). While bisphosphonates are generally well-tolerated, they can cause side effects in some people, such as gastrointestinal issues (like nausea, heartburn, or stomach pain), flu-like symptoms, and serious complications like osteonecrosis of the jaw (ONJ). ONJ is often referred to as BRONJ (Bisphosphonate-Related Osteonecrosis of the Jaw), which is ONJ that occurs in patients who have been treated with bisphosphonates (3), or MRONJ (Medication-Related Osteonecrosis of the Jaw), which is a broader term that encompasses ONJ associated with not only bisphosphonates but also other medications (4), such as denosumab, a monoclonal antibody that inhibits RANKL (Receptor Activator of Nuclear Factor Kappa-B Ligand) (1), a key factor in osteoclast formation, function, and survival, and antiangiogenic agents. ONJ is associated with bisphosphonates, particularly when administered intravenously at higher doses, often in the treatment of cancer that has spread to the bone (metastatic bone disease). ONJ is characterized by the death of bone tissue in the jaw, leading to symptoms such as exposed bone in the mouth, pain and swelling.

## Mechanisms underlying BRONJ

The exact mechanism by which bisphosphonates contribute to ONJ is not fully understood, although studies suggest that the disruption in the normal bone remodeling process, leading to impaired bone healing and the accumulation of dead bone tissue, might depend on bisphosphonates-mediated inhibition of both osteoclast differentiation and angiogenesis (3, 4). This can result in the persistence of non-healing lesions and the development of exposed bone in the mouth, which are characteristic features of ONJ. Furthermore, osteoclast inhibition by bisphosphonates may impair the ability of the jawbone to respond to injury or infection, further contributing to the development of the disease. Additionally, the jawbone is subject to microdamage and trauma from everyday activities like chewing and dental procedures, which can exacerbate the effects of bisphosphonates on bone healing in this region, thereby triggering the onset of ONJ. Experimental data show that bisphosphonates do not only deregulate osteoclast differentiation, but also negatively condition macrophage function (5). One of the primary functions of macrophages is to phagocytize dead cells and necrotic tissue. In ONJ, impaired macrophage function can lead to the accumulation of necrotic bone and delayed healing. Macrophages are involved in tissue repair and regeneration, they secrete growth factors like Transforming Growth Factor β (TGF-β) and Vascular Endothelial Growth Factor (VEGF), which promote angiogenesis and tissue repair. In ONJ, insufficient macrophage response can hinder these processes, leading to poor wound healing and prolonged exposure of necrotic bone.

## New local therapies targeting osteoclast differentiation in ONJ

While osteoclast inhibition by bisphosphonates is beneficial, at the systemic level, for managing conditions like osteoporosis and bone metastases, it can contribute, locally, to the pathogenesis of ONJ by disrupting normal bone remodeling processes in the jaw. In this regard, the development of treatments allowing to locally revert the inhibition of osteoclast differentiation in the jaw of bisphosphonates patients might be of particular interest. In literature there are three publications directly or indirectly suggesting three different approaches aimed to potentiate osteoclast differentiation or mitigate bisphosphonates activity, that might be further developed in order to locally target BRONJ. They are respectively based on the inhibition of Glycogen synthase kinase-3 beta (GSK3β) (6), geranyl-geraniol (GG) administration (7) or local use of magnesium (Mg) (8–10). GSK3β is a highly conserved serine/threonine kinase active in different signal transduction pathways. Several studies, that were summarized and discussed in a recent review (6), evidenced that inhibition of GSK3β, obtained by phosphorylation or down-regulation, in the presence of the cytokines macrophage colony- stimulating factor (M-CSF) and nuclear factor Kappa B ligand (RANKL) favors *in vitro* differentiation of osteoclast precursors into osteoclasts. Although the target of these studies was the comprehension of the role of GSK3β in bone remodeling with regards to its role in osteoporosis, these observations suggest that GSK3β inhibition, obtained for instance by using lithium chloride (LiCl), might be an interesting putative target also for the therapy of ONJ. Nevertheless, the same review observes that in literature there are discrepancies between *in vitro* and *in vivo* experiments, making this option very premature and in need of further studies. GG is a metabolite in the mevalonate metabolism, which is strongly involved in the effect of bisphosphonates (11, 12). GG is a product of reactions catalyzed by farnesyl pyrophosphate synthase (FPPS). FPPS catalyzes the synthesis of farnesyl pyrophosphate (FPP), a key intermediate in the mevalonate pathway. This pathway is essential for the production of cholesterol, isoprenoids, and other important molecules involved in cell membrane integrity, protein prenylation, and bone metabolism (11, 12). By competing with pyrophosphate (PP) residues in the active site of FPPS, bisphosphonates disrupt the mevalonate pathway, leading to reduced production of FPP and subsequent downstream metabolites like GG (11, 12). This inhibition impairs the prenylation of small GTPase signaling proteins, and leads to decreased osteoclast activity and increased osteoclast apoptosis. While this reduces bone resorption and strengthens bones, it also disrupts normal bone remodeling and repair processes. Impaired osteoclast function and bone remodeling can contribute to the accumulation of microdamage and necrotic bone, especially in the jaw, which has a high rate of bone turnover. It has been demonstrated that by adding GG to *in vitro* cell cultures it is possible to restore the downstream mevalonate pathway, therefore mitigating the apoptotic and anti-differentiative effect of bisphosphonates on osteoclast precursors (7). The third approach suggests that specific concentrations of Mg are capable to induce osteoclast differentiation and, moreover, that this effect is greatly enhanced by the presence of the bisphosphonate zoledronate (8). This approach, that was described in detail also in two patents (Italian Patent: Title “Therapy of Osteonecrosis of the Jaw”, Inventor Alexis Grande, Priority Number 102023000002301, Priority Date 10/02/2023, https://www.knowledge-share.eu. Patent Cooperation Treaty, PCT: Title “Prevention and Treatment of Maxillary Osteonecrosis”, IA number: PCT/IB2024/051172, International filing date: 08/02/2024), is particularly interesting, since Mg is currently used as a dietary supplement and its topic use is free of toxicity. Another drug that was tested to treat ONJ is Teriparatide (13), a recombinant form of parathyroid hormone (PTH), specifically the first 34 amino acids of the human PTH (PTH 1-34). Teriparatide was administered systemically and proved to stimulate bone formation. Its local use might also be tested, although the protein nature of the molecule might reduce its availability in the cell and overall efficacy.

## Discussion

**Figure 1:**
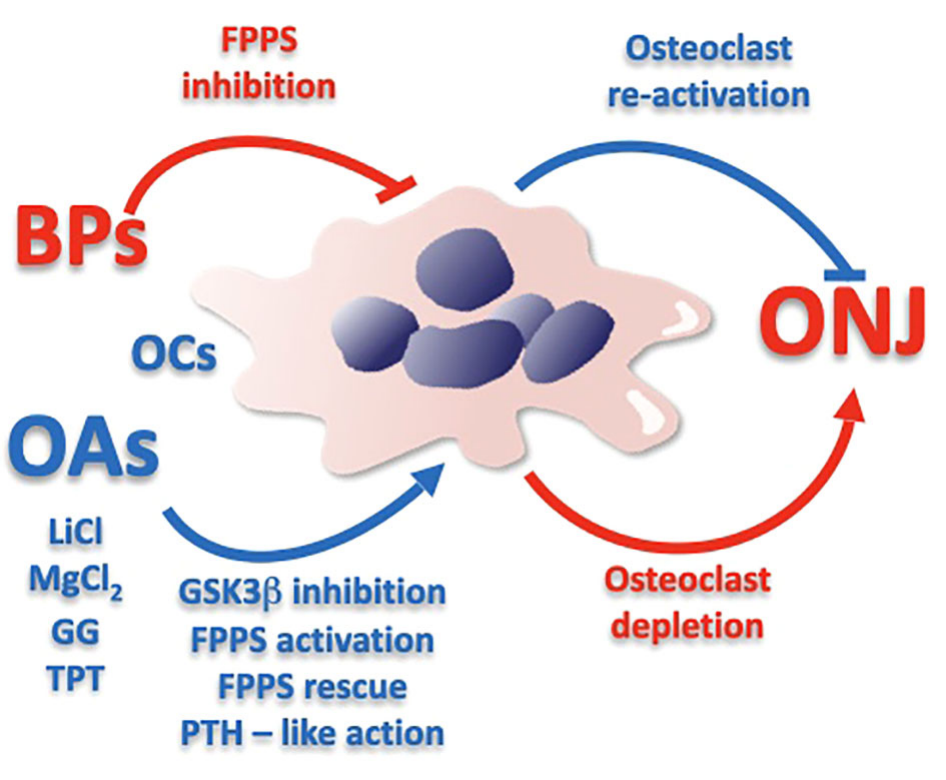
Schematic representation of the potential effect that might be determined by Osteoclastogenic Agents in the prevention and treatment of Osteonecrosis of the Jaw. BPs, Bisphosphonates; OAs, Osteoclastogenic Agents; ONJ, Osteonecrosis of the Jaw; OCs, Osteoclasts; LiCl, Lithium Chloride; MgCl_2_, Magnesium Chloride; GG, Geranyl – Geraniol; TPT, Teriparatide; GSK3β, Glycogen synthase kinase-3 beta; FPPS, Farnesyl Pyrophosphate Synthase; PTH, Parathyroid hormone. Arrow and hammer-headlines respectively mean stimulation and inhibition.

Since systemic treatment with bisphosphonates or denosumab has proven its efficacy in the treatment of life-threatening conditions, the development of local therapies for ONJ that do not interfere with the systemic treatment might be of interest (**Table 1**). Topical therapies based on GG and Mg might indeed prove to be effective in mitigating the local collateral symptoms that bisphosphonates trigger in the jaw, while leaving the systemic effect intact. Both the GG and Mg approach act on osteoclast differentiation (**Figure 1**): GG restores the mevalonate pathway, while Mg seems to be active at different levels. In particular, supra-physiological concentrations of Mg are capable of promoting osteoclast differentiation in the presence of bisphosphonates (8); Mg is capable to autonomously promote macrophage and osteoclast differentiation (8, 10) and, in addition, Mg plays a role in cellular energy metabolism, DNA and protein synthesis, and has anti-inflammatory effects thereby supporting overall bone health and cellular functions, providing a favorable environment for bone repair and regeneration (14). The mechanism underlying the ability of Mg to enhance osteoclast differentiation at the presence of bisphosphonates has not been clarified, but it is possible to hypothesize that the interaction of PP with the active catalytic site of the FPPS enzyme is mediated by Mg and it is therefore possible that, through a conformational change mechanism, an increase in Mg concentration might determine an opposite effect on the recruitment of PP or bisphosphonates, favoring the former and inhibiting the latter. This mechanism would allow to hypothesize that both GG and Mg might be useful in the treatment of BRONJ, given the specific involvement of the mevalonate pathway. Since Mg seems to be capable of promoting autonomously both macrophage and osteoclast differentiation (15), local treatment with Mg in various forms (solution, gel or other) might be useful also in the treatment of MRONJ, for instance denosumab-related. This possibility should be experimentally tested in a RANKL-dependent osteoclast differentiation model. It would also be imaginable to develop a combined treatment for BRONJ with GG and Mg that might combine a synergistic or additive effect of the two on osteoclast differentiation with the additional positive effect of Mg on macrophage differentiation and inflammation.

**Table 1:** List of Osteoclastogenic Agents which are potentially available for the prevention and treatment of ONJ.

Osteoclastogenic Agent	Active Concentration	Reference number
LiCl	10 mM	(6)
GG	10 μM	(7)
MgCl_2_	10 mM	(8–10)
Teriparatide	Undetermined	(13)

MgCl_2_, Magnesium Chloride; GG, Geranyl – Geraniol; LiCl, Lithium Chloride; Teriparatide.

## Author contributions

TZ-M: Conceptualization, Funding acquisition, Writing – original draft, Writing – review & editing. SR: Investigation, Writing – review & editing. LC: Investigation, Writing – review & editing. ELo: Investigation, Visualization, Writing – review & editing. ELe: Investigation, Writing – review & editing. AG: Conceptualization, Supervision, Writing – review & editing.
